# Crohn’s disease: research progress in decoding pathogenic multi-network and precision management of artificial intelligence radiomics

**DOI:** 10.3389/fimmu.2026.1774889

**Published:** 2026-03-12

**Authors:** Wumiao Zhang, Hua Xie, Shuyan Ying, Xueliang Zeng, Xiaomin Liao, Shengyan Hu, He Zeng, Qinghua Zou, Dingcheng Zeng, Fan Meng

**Affiliations:** 1 The First Clinical Medical College of Gannan Medical University, Ganzhou, Jiangxi, China; 2 Department of Gastroenterology, The First Affiliated Hospital of Gannan Medical University, Ganzhou, Jiangxi, China; 3 Department of Pharmacy, The First Affiliated Hospital of Gannan Medical University, Ganzhou, Jiangxi, China; 4 Department of Pathology, The Third Affiliated Hospital of Wenzhou Medical University, Wenzhou, Zhejiang, China

**Keywords:** artificial intelligence, Crohn’s disease, imaging biomarkers, multi-network pathogenesis, radiomics

## Abstract

Crohn’s disease (CD) is a chronic, relapsing inflammatory bowel disease characterized by transmural inflammation. Its clinical presentation and disease course are highly heterogeneous across individuals, and the global disease burden continues to rise. Although biomarkers such as fecal calprotectin and anti–Saccharomyces cerevisiae antibodies (ASCA), together with computed tomography enterography (CTE)/magnetic resonance enterography (MRE) and endoscopy, play central roles in diagnosis and longitudinal monitoring, important unmet needs remain. In particular, current approaches show limited reproducibility and insufficient phenotypic granularity for stratifying transmural inflammation, mesenteric involvement, and fibrostenotic disease, as well as for predicting therapeutic response and surgical risk. In this review, we adopt a multi-network pathogenic framework—encompassing genetic susceptibility, barrier dysfunction, microbial dysbiosis, immune-driven inflammation, fibrotic remodeling, and mesenteric inflammation with adipose remodeling—to delineate how these interconnected processes shape intestinal and mesenteric imaging phenotypes. We then focus on AI-enabled radiomics in CTE/MRE, summarizing key workflows for phenotype quantification, feature extraction, and model development, and highlighting its potential as an imaging biomarker across major clinical applications, including diagnosis and differential diagnosis, assessment of inflammatory activity, fibrosis stratification, prediction of treatment response, and surgical risk management. Importantly, rather than treating radiomics as a purely predictive “black box,” we organize current evidence within a mechanism-to-phenotype framework that links multi-network pathobiology and the histology/microenvironment to CTE/MRE imaging phenotypes and downstream radiomic signatures, thereby providing a biologically anchored basis for interpretation and model design. Finally, we discuss major challenges to clinical translation, including inter-center variability, differences in image acquisition and reconstruction, segmentation uncertainty, feature robustness, limited external validation, and clinical interpretability. We further outline a feasible roadmap for integrating radiomics with immunologic multi-omics to build a translatable evidence framework that supports precision management in CD.

## 1 Introduction

Crohn’s disease (CD) is a major form of inflammatory bowel disease (IBD), characterized predominantly by chronic, relapsing, transmural inflammation (1). Its clinical presentation and disease course are highly heterogeneous: patients may exhibit inflammatory, stricturing, or penetrating phenotypes, frequently accompanied by mesenteric involvement and bowel-wall remodeling (2). These features drive recurrent flares and increase rates of hospitalization and surgery, substantially compromising quality of life and imposing a considerable socioeconomic burden (1). With both incidence and prevalence continuing to rise worldwide (3), several priorities have become central to contemporary CD management: achieving early diagnosis and accurate differential diagnosis, dynamically monitoring disease activity, identifying fibrotic strictures, and reliably predicting therapeutic response and surgical risk (1).

Serum and fecal biomarkers, endoscopy, and cross-sectional imaging are all important adjuncts in the diagnosis and assessment of CD; however, each has distinct inherent limitations. Fecal calprotectin, C-reactive protein, and serologic antibody profiles (e.g., anti-Saccharomyces cerevisiae antibodies [ASCA]) provide useful surrogates of inflammatory activity (2, 4). However, they have limited capacity to capture the depth and extent of transmural inflammation, mesenteric inflammatory responses, or fibrotic remodeling, and their levels can be influenced by multiple non-specific factors (5, 6). Endoscopy enables direct visualization of mucosal lesions and permits histologic sampling, yet it cannot comprehensively assess the small bowel or extraintestinal/mesenteric manifestations, and it remains suboptimal for evaluating transmural disease and deep fibrosis. Computed tomography enterography (CTE) and magnetic resonance enterography (MRE) offer an integrated view of the bowel wall and extramural structures and are invaluable for assessing wall thickening, mural hyperenhancement, ulceration, strictures, and mesenteric vascular engorgement (7). Nevertheless, two major bottlenecks persist in routine practice. First, many imaging signs are still described in semi-quantitative or subjective terms and are therefore sensitive to acquisition parameters, reader expertise, and inter-center variability (8). Second, conventional imaging assessment remains insufficient to characterize the biological heterogeneity of CD, particularly with respect to discriminating inflammation from fibrosis, predicting treatment response and relapse risk, and establishing robust, generalizable quantitative metrics that can be reproducibly validated across centers (9).

Against this backdrop, we contend that improving the precision of CD management should begin with a mechanistic discussion of its multi-network pathobiology. From a mechanistic standpoint, CD is not driven by a single pathway but represents a multi-network disorder shaped by the interplay of genetic susceptibility, epithelial barrier dysfunction, gut microbiome dysbiosis, activation of immune-inflammatory circuits, and fibrotic remodeling (10). These processes not only determine the magnitude and distribution of mucosal inflammation but also induce measurable structural and functional alterations across the layered architecture of the bowel wall and the mesenteric microenvironment. Such changes can manifest on cross-sectional imaging—particularly CTE and MRE—as a spectrum of recognizable imaging phenotypes (11). Accordingly, advancing imaging phenotypes beyond conventional qualitative descriptions toward reproducible, validated quantitative metrics may open a new, noninvasive avenue for CD management, enabling improved diagnosis, precision phenotypic stratification, assessment of inflammatory activity, fibrosis/fibrostenosis stratification, and more informed treatment decision-making.

In recent years, advances in artificial intelligence (AI) and radiomics have provided a feasible route toward this goal. Radiomics enables high-throughput feature extraction from medical images, converting subtle information—such as texture, shape, intensity distributions, and spatial heterogeneity that may be imperceptible to the human eye—into quantitative, high-dimensional descriptors (12). When coupled with machine learning or deep learning, these features can be leveraged to develop predictive models with the potential to yield imaging biomarkers that reflect underlying pathobiological processes (13). Compared with conventional qualitative assessment or scoring systems, radiomics may improve clinical decision-making across several pivotal scenarios, including discriminating active inflammation from fibrotic strictures, quantifying transmural and mesenteric involvement, predicting response to biologics such as anti-TNF agents, and identifying high-risk patients who may benefit from treatment escalation or earlier surgical intervention (14–16). At the same time, radiomics research faces substantial challenges, including limited reproducibility, variability introduced by segmentation strategies, overfitting, insufficient external validation, and persistent barriers related to clinical interpretability and real-world implementation (17, 18). These issues underscore the need for systematic synthesis and critical appraisal within standardized, multicenter validation frameworks.

**Figure 1:**
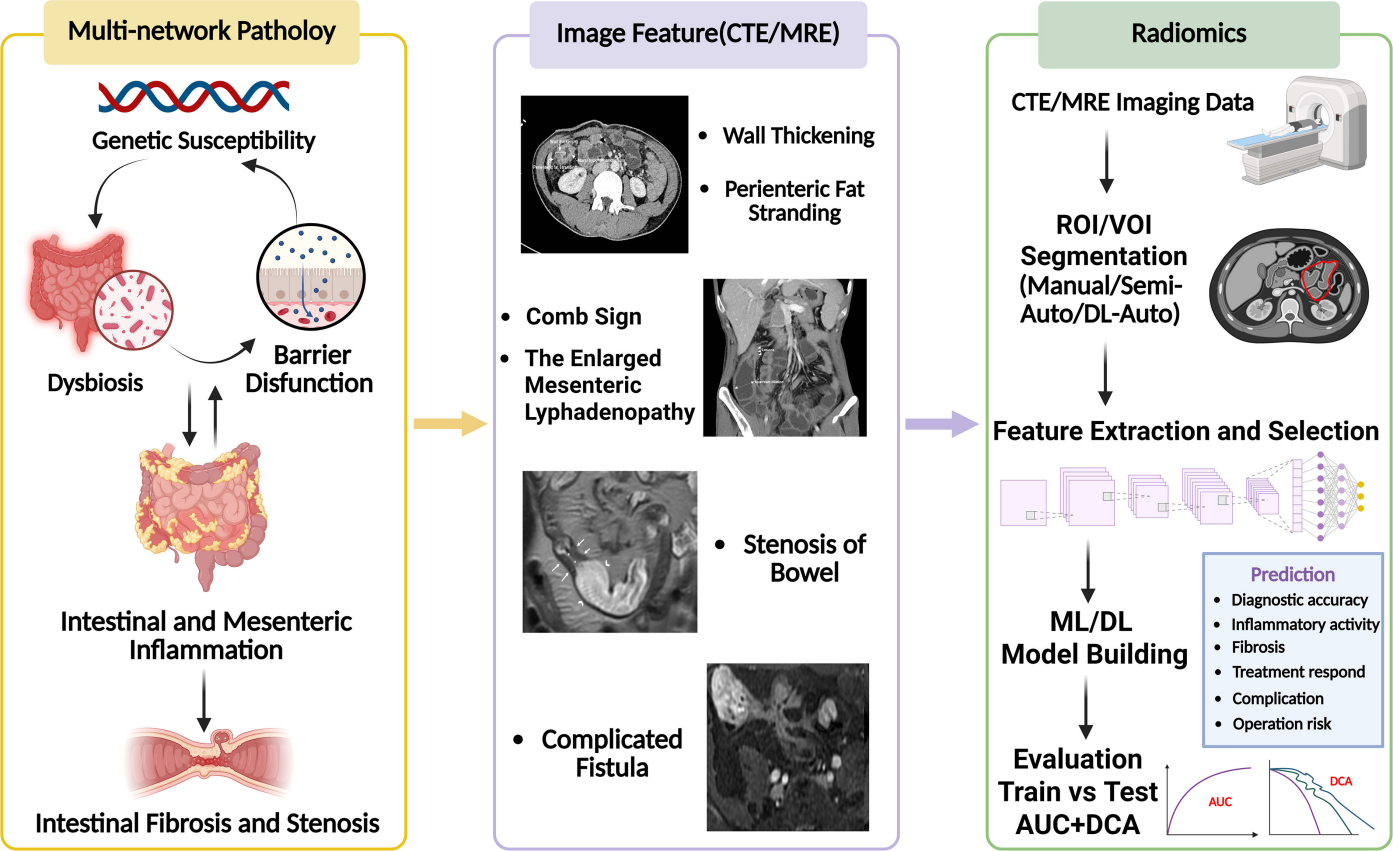
Conceptual framework of this review.

Accordingly, this review is organized around a mechanistic cascade—genetic susceptibility, barrier dysfunction, microbial dysbiosis, immune-driven inflammation, fibrotic remodeling, and mesenteric inflammation with adipose remodeling—and describes how these interconnected processes collectively shape intestinal and mesenteric imaging phenotypes. We then synthesize the typical radiomics workflows in CTE/MRE, summarize the principal clinical applications and current evidence, and critically examine the key methodological factors that determine model robustness, generalizability, and clinical translatability. Finally, we outline feasible strategies for integrating radiomics with immunologic multi-omics, with the aim of moving CD care from experience-based assessment toward reproducible, quantitatively validated decision-making. The overarching framework of this review is summarized in **Figure 1**.

## 2 Multi-network pathobiology shapes histologic and imaging phenotypes of the bowel wall and mesentery in Crohn’s disease

**Figure 2:**
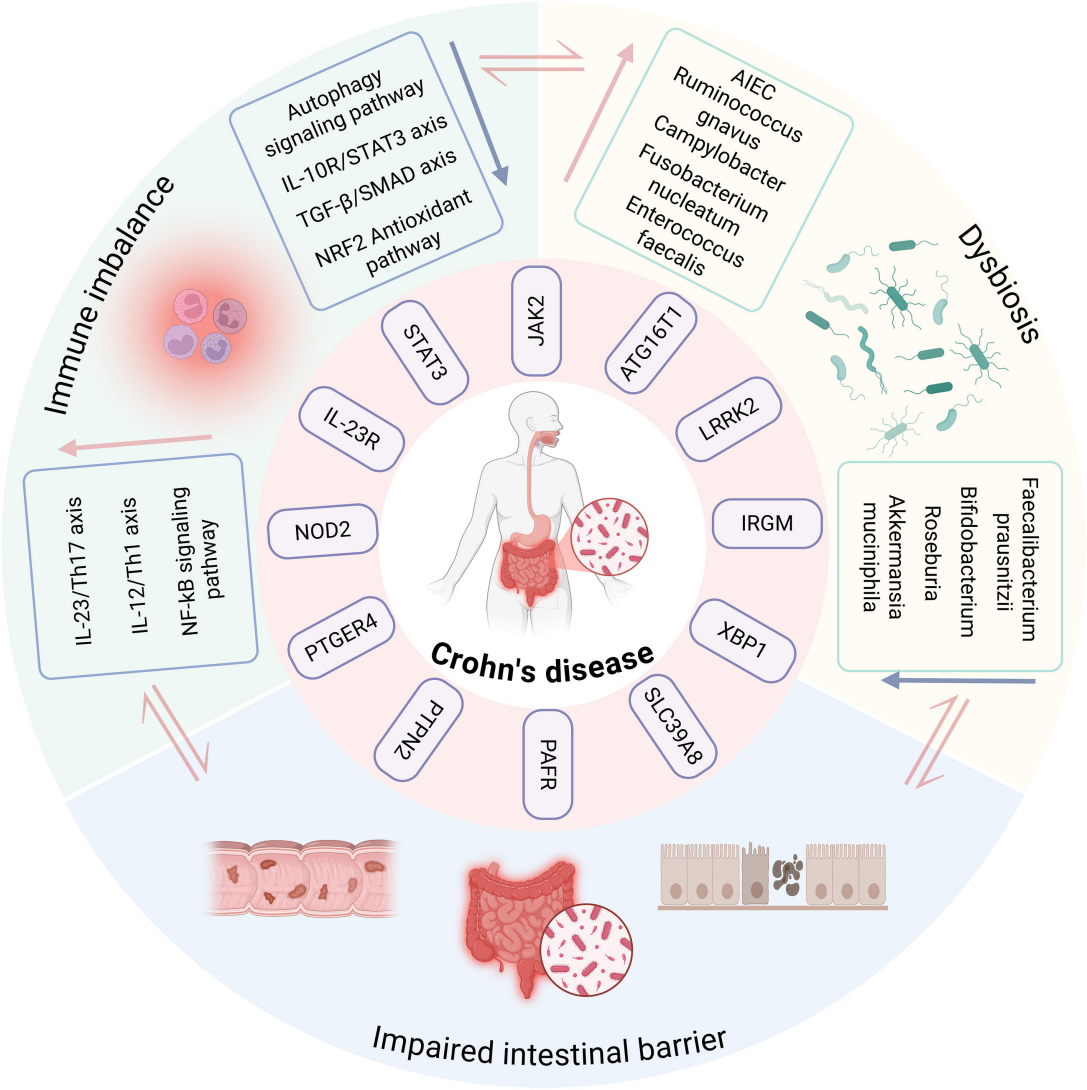
Mechanism of Crohn's disease. The interaction between susceptibility genes, immune imbalance, dysbiosis, and intestinal barrier damage leads to the occurrence of Crohn's disease.

CD is best understood as a multi-network disorder rather than the product of a single pathogenic pathway. Genetic susceptibility, epithelial barrier dysfunction, and gut microbiome dysbiosis interact to initiate and perpetuate immune-inflammatory circuits, ultimately driving transmural injury and—over time—fibrotic remodeling and structural damage (19). As summarized in **Figure 2**, these modules are tightly interconnected and mutually reinforcing, providing a mechanistic framework for interpreting downstream histologic and imaging phenotypes.

### 2.1 Genetic susceptibility shapes the immune set point and contributes to phenotypic heterogeneity in CD

Genetic susceptibility does not predetermine a specific clinical phenotype in CD; rather, it modulates how the host senses microbial cues and sets the “sensitivity threshold” of immune activation (20–22). In turn, this altered immune set point can influence disease location, behavior (inflammatory, stricturing, or penetrating), and trajectories of progression. Genetic studies indicate that CD-associated risk loci are primarily enriched in three functional domains: (i) microbial recognition and innate immunity (e.g., NOD2, CARD9); (ii) epithelial intrinsic defense, including the autophagy–endoplasmic reticulum stress axis (e.g., ATG16L1, IRGM, XBP1); and (iii) inflammatory maintenance pathways centered on IL-23/Th17 signaling and NF-κB activation (e.g., IL23R-related signaling) (23, 24). Dysfunction across these pathways can impair antigen clearance, weaken mucosal defenses, and sustain pro-inflammatory signaling, thereby promoting deep fissuring ulceration and transmural inflammation. Over time, recurrent injury and unresolved inflammation may drive smooth muscle hypertrophy and extracellular matrix deposition, increasing the risk of fibrostenotic and penetrating complications. Notably, genotype–phenotype studies have linked variants such as those in NOD2 to ileal involvement and a stricturing disease behavior, providing an upstream mechanistic basis for downstream imaging observations—namely, inter-individual differences in transmural inflammatory signals and stricture burden (20).

### 2.2 Barrier dysfunction and microbial translocation sustain a pro-inflammatory mucosal microenvironment

**Figure 3:**
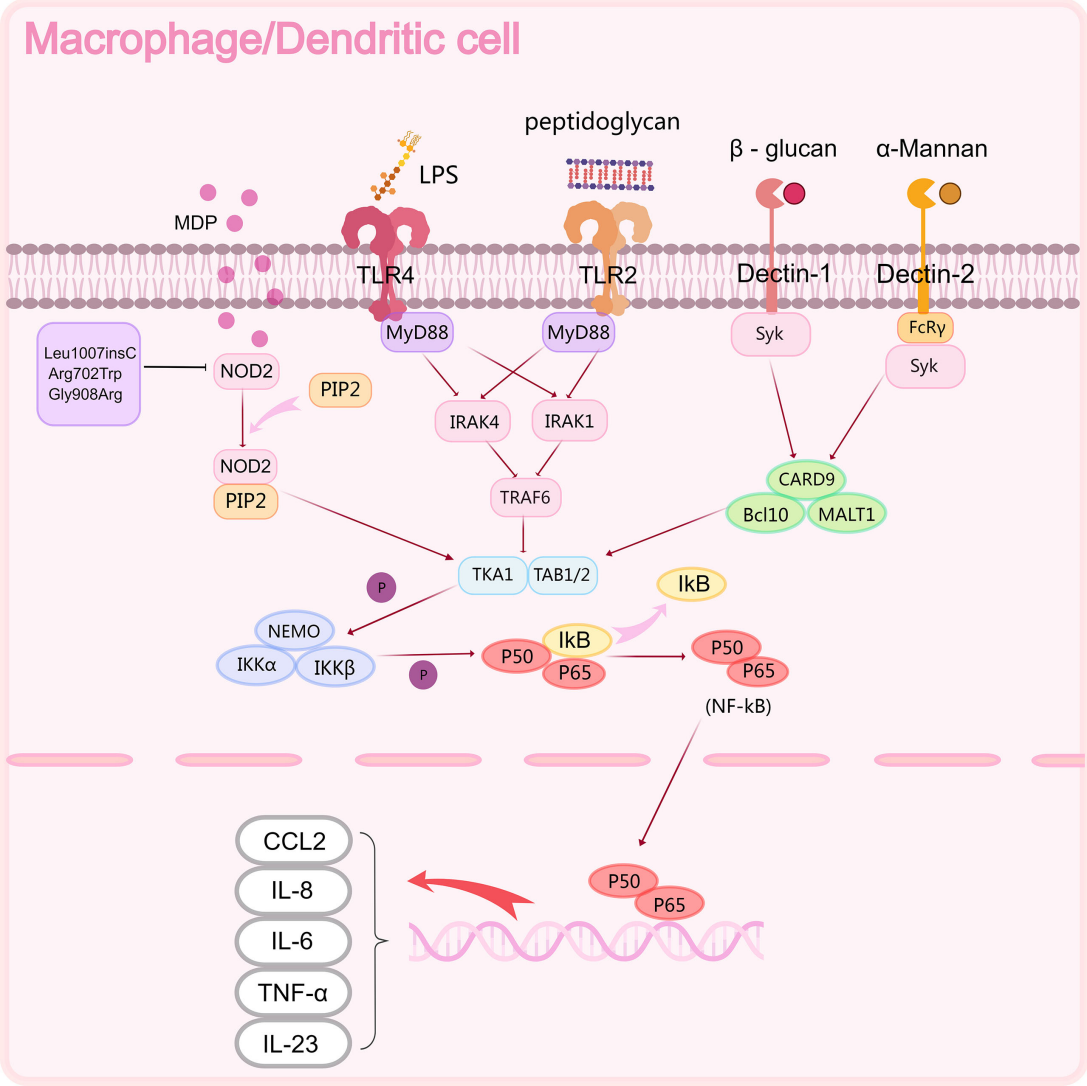
Activation of NF-κB signaling pathway in Crohn's disease. The NF kB signaling pathway of macrophages and dendritic cells can be activated by various components of microorganisms (such as MDP, LPS, β - glucan, α - Mannan), ultimately producing multiple inflammatory factors (IL-6, IL-8, IL-23) and chemokines (CCL2).

Epithelial barrier dysfunction is a central “initiator and amplifier” of chronic inflammation in CD. Importantly, the key defect is not simply focal mucosal breaks, but a coordinated failure of the mechanical barrier, chemical barrier, and immune surveillance (25, 26). At the mechanical barrier level, the tight-junction complex—maintained by transmembrane proteins (e.g., claudins, occludin, JAM-A) and scaffolding proteins (e.g., ZO-1/TJP1)—regulates paracellular permeability. Disrupted expression or mislocalization of these components increases epithelial permeability, allowing luminal microbes and their products to translocate into the lamina propria (27). At the chemical barrier level, dysfunction of ileal Paneth cells and goblet cells can reduce antimicrobial peptides (e.g., α-defensins, lysozyme) and compromise the mucus layer and MUC2-mediated “spatial segregation,” thereby facilitating bacterial adherence and direct microbe–epithelium contact, which further exacerbates barrier injury (28, 29). With respect to immune sensing, translocated pathogen-associated molecular patterns (PAMPs) chronically activate pattern-recognition receptors on macrophages and dendritic cells. For example, TLR4 senses lipopolysaccharide (LPS), TLR2 responds to peptidoglycan-related bacterial components, Dectin-1 and Dectin-2 recognize fungal β-glucans and α-mannans, respectively, and intracellular muramyl dipeptide (MDP) is detected by NOD2 (30). These upstream cues signal predominantly through the MyD88 (TLR2/4), NOD2, and CARD9 pathways, converging on NF-κB activation and driving the transcription of pro-inflammatory cytokines and chemokines, including TNF-α, IL-6, IL-8 (CXCL8), CCL2, and IL-23 (31)(**Figure 3**). The resulting recruitment of neutrophils and monocytes—together with endothelial activation and increased vascular permeability—promotes intramural edema and accelerates ulcer progression (32). In terms of repair, imbalanced epithelial regeneration and increased cell death impair stable wound healing, creating a self-reinforcing loop of “barrier disruption–inflammation–recurrent injury.” (33).

Histologically, these processes manifest as mucosal ulceration and inflammatory infiltrates, with extension to transmural edema and perienteric exudation (34). When inflammation extends beyond the mucosa and elicits a prominent transmural response, CTE/MRE typically demonstrates concomitant bowel-wall thickening and layered (stratified) enhancement, often accompanied by perienteric fat stranding and mesenteric hypervascularity (e.g., the comb sign) (35). Collectively, these findings translate the “barrier disruption–microbial translocation–transmural inflammation” cascade into a recognizable imaging phenotype.

### 2.3 Dysbiosis reshapes inflammatory programs and amplifies clinical heterogeneity

Accumulating evidence suggests that dysbiosis is not only a permissive background for chronic inflammation but may also help explain why immune-inflammatory programs and clinical phenotypes vary markedly across patients (36, 37). For example, Britton and colleagues transplanted fecal microbiota from multiple IBD donors (including CD and UC) into germ-free mice and observed that IBD-derived communities induced higher intestinal Th17 responses accompanied by lower RORγt^+^ Treg frequencies; importantly, the Th17/Treg balance differed substantially across donors, supporting the concept that functional differences among donor microbiotas can shape distinct immune set points (37).

Gut microbial dysbiosis in CD is commonly characterized by a reduction in Firmicutes and an expansion of Proteobacteria (38). One repeatedly observed feature of CD dysbiosis is the depletion of short-chain fatty acid (SCFA)–producing taxa (39). SCFAs—particularly butyrate—serve as a major energy source for colonocytes and contribute to barrier maintenance by supporting tight-junction integrity and mucus-layer function (40, 41). Beyond epithelial support, SCFAs also promote immune tolerance through G protein–coupled receptor signaling and epigenetic regulation, favoring Treg-associated programs and restraining excessive pro-inflammatory responses (42). Thus, reduced SCFA availability can weaken epithelial repair and barrier resilience, facilitate bacterial translocation, and tip the local immune milieu toward sustained inflammation and tissue injury. In parallel, enrichment of adherent-invasive strains can enhance mucosal adherence and invasion, prolong antigenic stimulation, and amplify innate and adaptive immune activation, resulting in increased production of inflammatory mediators such as IL-23, IL-1β, and IL-6 (43). Compared with bacterial dysbiosis, alterations in the mycobiome and virome may further modulate inflammatory networks through cross-kingdom interactions and interferon-related pathways, potentially contributing to inter-individual differences in treatment response (44).

At the metabolic level, tryptophan and bile acid pathways provide additional mechanistic links between dysbiosis, epithelial repair, and immune polarization (45). With respect to tryptophan metabolism, commensal microbes generate indole derivatives (e.g., IAA, IPA, IAld) that act as aryl hydrocarbon receptor (AHR) ligands, activating AHR signaling in epithelial cells and immune subsets such as ILC3s (46–48). Through the AHR–IL-22 axis, these metabolites promote epithelial regeneration and mucosal defense programs (including mucus and antimicrobial responses), thereby reinforcing barrier homeostasis and limiting bacterial translocation (48). When dysbiosis reduces indole production—or when inflammation diverts host tryptophan toward the kynurenine pathway—protective AHR signaling diminishes, facilitating a self-sustaining cycle of impaired repair and persistent innate immune activation (49). In the bile acid pathway, the gut microbiota reshapes the bile acid pool through deconjugation and secondary bile acid production. These bile acids act as signaling molecules that engage the nuclear receptor FXR and the membrane receptor TGR5 (50). FXR signaling supports intestinal homeostasis and barrier function and, via the gut–liver axis, modulates bile acid pool composition in ways that can secondarily influence microbial community structure (51). In parallel, TGR5 can suppress apoptosis in intestinal epithelial cells (IECs) by engaging the cAMP/PKA pathway and downstream c-FLIP/JNK signaling (52). Conversely, when bile acid composition is perturbed or FXR/TGR5-mediated protective signaling is insufficient, barrier vulnerability and permeability increase, facilitating PAMP translocation and mucosal immune activation. This immune activation can further disrupt microbial composition and bile acid biotransformation, creating a self-reinforcing “microbiota–bile acids–receptor signaling–barrier/inflammation” feedback loop that may help sustain chronic disease.

In imaging, while there is no distinct indicator of intestinal microecological imbalance, the resultant barrier vulnerability and transmural inflammation can frequently manifest as an identifiable phenotypic combination. Recent research in 2025 has shown that the metabolites of intestinal microflora, specifically N-acetylneuraminic acid and guanine acetic acid, were associated with morphological alterations of intestinal MRE and the intensity of inflammation (53). This discovery offers initial evidence for the connection among gut microbiota, metabolites, and imaging.

### 2.4 Immune-inflammatory networks drive transmural injury and disease-behavior progression

**Figure 4:**
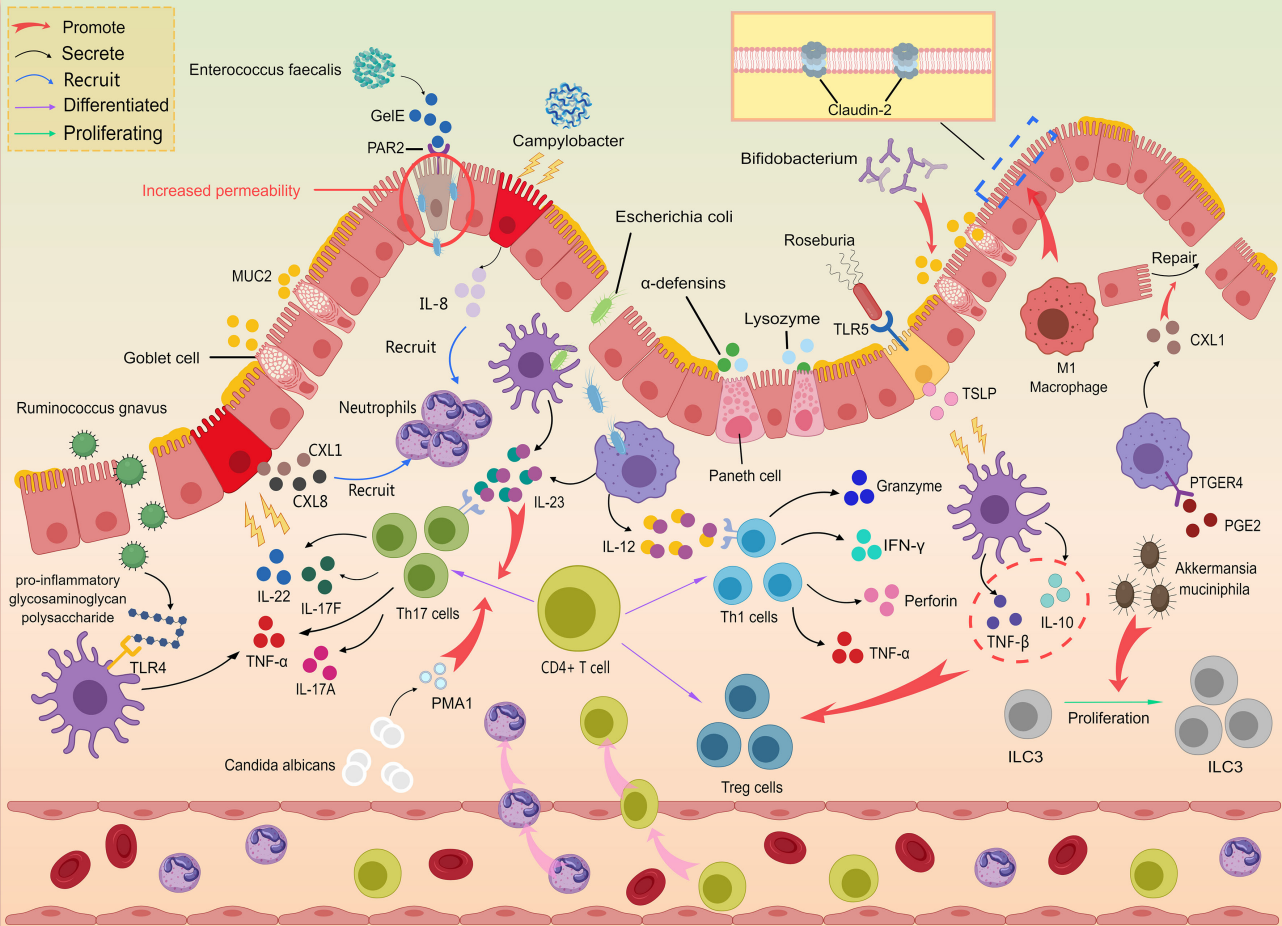
Schematic overview of the immune-inflammatory network in Crohn’s disease. Microbial stimuli and epithelial barrier dysfunction jointly initiate and sustain mucosal immune activation in Crohn’s disease. The figure summarizes how epithelial cells (including Paneth and goblet cells), innate immune sensing pathways, and immune cell subsets (e.g., macrophages, Th17/Th1/Treg cells, and ILC3) interact through cytokine-mediated crosstalk. It highlights the dynamic imbalance between pro-inflammatory amplification (e.g., IL-23–Th17-related signaling) and regulatory/repair pathways (e.g., Treg/IL-10-associated responses and barrier repair), which together shape the persistent mucosal and transmural inflammatory microenvironment.

In this setting, increased epithelial permeability promotes the persistent translocation of microbial products, thereby reinforcing the barrier–microbiota–immune crosstalk that amplifies downstream inflammatory cascades (**Figure 4**).A defining pathological feature of CD is that inflammation extends beyond the mucosa into deeper layers, culminating in transmural injury and, in many patients, luminal narrowing (54). The intensity and depth of this process are shaped by persistently activated innate and adaptive immune programs. NF-κB signaling not only amplifies pro-inflammatory cytokine cascades but also promotes endothelial activation and upregulation of adhesion molecules, facilitating leukocyte extravasation and increasing vascular permeability—thereby exacerbating intramural edema and ulcer progression (55). In parallel, the IL-23/Th17 axis sustains chronic inflammatory circuits and drives continued neutrophil recruitment via chemokine programs, making tissue damage difficult to contain (56). Concurrently, proteases and matrix metalloproteinases (MMPs) released by macrophages and neutrophils promote extracellular matrix degradation and tissue “tunneling,” providing a direct pathological basis for fissuring ulcers, sinus tracts, and fistula formation (57). Dysregulated angiogenesis and impaired lymphatic drainage further alter immune-cell trafficking and interstitial fluid dynamics, facilitating extension of inflammation into the perienteric tissues and mesentery. Against this histopathologic backdrop, CTE/MRE detection of transmural inflammation can be viewed as an integrated readout of several measurable processes: altered perfusion and permeability (contrast enhancement), increased free water content (T2 hyperintensity), increased cellularity (restricted diffusion on diffusion-weighted imaging [DWI] with lower apparent diffusion coefficient[ADC]), disruption of mural structural continuity (ulcers, sinus tracts, fistulas), and perienteric reactions (fat stranding and serosal inflammatory changes) (8). Notably, prior studies have shown that MRE can identify occult sinus tracts and related transmural injury phenotypes, and that such findings are associated with a higher subsequent risk of penetrating complications—supporting the concept that imaging can capture clinically meaningful consequences along the trajectory from transmural inflammation to structural “tunneling” (58).

### 2.5 Fibrotic remodeling consolidates inflammatory injury into stricture-related structural damage

Fibrosis represents a pivotal transition in CD from active inflammation to irreversible structural damage, and it is a major determinant of therapeutic strategy and long-term prognosis (59). Fibrosis in CD reflects a maladaptive repair response driven by chronic, relapsing inflammatory injury. Persistent mucosal disruption and transmural inflammation maintain intestinal fibroblasts, myofibroblasts, perivascular cells and smooth muscle–lineage cells in a sustained activated state, progressively shifting them toward a phenotype dominated by extracellular matrix (ECM) production and contractility (60). Among pro-fibrotic pathways, transforming growth factor–β (TGF-β) is widely regarded as a central hub. Through canonical TGFBR1/2–SMAD2/3 signaling, TGF-β promotes transcription of key fibrotic and contractile genes, including COL1A1, COL3A1, FN1, and ACTA2, and cooperates with non-canonical branches such as PI3K–AKT–mTOR and RhoA–ROCK to enhance cell survival, protein synthesis, and assembly of the contractile apparatus (61, 62). Platelet-derived growth factor (PDGF) primarily acts via ERK and AKT signaling to drive fibroblast proliferation and migration, expanding the pool of ECM-producing cells (63). Connective tissue growth factor (CTGF/CCN2), a key mediator integrating TGF-β signaling with mechanical cues, further reinforces collagen deposition and tissue stiffening. ECM accumulation reflects not only increased synthesis but also impaired clearance. An imbalance between matrix metalloproteinases and their inhibitors (MMP/TIMP) and PAI-1–mediated suppression of fibrinolysis reduces matrix turnover, whereas LOX/LOXL-driven crosslinking renders collagen more compact and resistant to degradation (64). As matrix stiffness increases, mechanotransduction pathways—including integrin–FAK–RhoA/ROCK and YAP/TAZ—become persistently activated, inducing pro-fibrotic programs (including CTGF) and establishing positive feedback (65, 66). This framework helps explain the clinical observation that, once established, strictures often enter a self-reinforcing remodeling trajectory. From an imaging perspective, fibrotic tissue is characterized by abundant collagen/ECM with relatively reduced cellularity and mobile water. Because T2-weighted MRE is particularly sensitive to free (mobile) water, fibrosis is often associated with relatively lower T2 signal intensity, less prominent submucosal edema, and delayed enhancement, reflecting slower diffusion and prolonged retention of contrast within the expanded interstitial compartment (67). Consistent with these principles, a prospective study by Coimbra and colleagues demonstrated that an MRE-derived quantitative metric (FibrosisMRE) correlates well with histologic fibrosis (68).

### 2.6 Mesenteric inflammation and fat remodeling contribute to disease persistence and adverse outcomes

Inflammation in CD is not confined to the bowel wall. Mesenteric inflammation and adipose remodeling constitute an important extraintestinal “zone of pathological extension” and have been associated with more complex disease behavior and adverse outcomes (69). Mechanistically, once intestinal inflammation increases barrier permeability, PAMPs, cytokines, and inflammatory exudates can more readily enter the perienteric and mesenteric compartments, leading to persistent activation of mesenteric adipocytes and the stromal–vascular fraction (70). This is followed by immune-cell infiltration and reshaping of the local immune landscape—most notably expansion of myeloid populations and a pro-inflammatory shift in macrophage polarization—with upregulated expression of TNF, IL-1β, IL-6, IL-23, and chemokines such as CCL2 and CXCL8 (71). These mediators sustain a pro-inflammatory microenvironment and continuously amplify leukocyte recruitment. In parallel, dysregulated adipokine signaling and extracellular matrix remodeling reduce the likelihood of inflammatory resolution, establishing a positive-feedback loop in which mesenteric adipose inflammation and bowel wall inflammation mutually reinforce one another (72). Imaging can capture these tissue and microcirculatory changes indirectly. On CTE/MRE, classic manifestations include engorged vasa recta (the “comb sign”), perienteric fat stranding, and serosal inflammatory reaction, collectively suggesting extension of inflammation beyond the bowel wall into the surrounding mesentery (35). In addition, abnormalities in lymphatic architecture and drainage may impair antigen clearance and immune-cell trafficking, thereby promoting persistence and outward spread of peripheral inflammation. Consistent with this concept, prior studies have reported that quantitative changes in the distribution of mesenteric/perienteric fat on CT correlate with disease activity, supporting the inclusion of mesenteric fat–based metrics—such as density and texture features—into imaging-driven stratification frameworks (73).

### 2.7 Convergent networks yield observable and quantifiable imaging phenotypes

Across the pathogenic continuum of Crohn’s disease—from genetic susceptibility to barrier disruption, microbial dysbiosis, immune-network activation, and ultimately fibrotic and mesenteric remodeling—these multi-network processes converge to produce a set.

## 3 The role and limitations of CTE/MRE in the assessment of CD

CTE and MRE are the cornerstone cross-sectional modalities for evaluating transmural disease and perienteric/mesenteric involvement in CD (74). Compared with endoscopy, which primarily reflects mucosal surface abnormalities, CTE/MRE provides comprehensive small-bowel coverage and characterizes mural stratification as well as perienteric and mesenteric structures, thereby aligning more closely with the hallmark pathology of transmural inflammation (75). CTE is rapid and offers high spatial resolution, making it particularly useful for acute presentations and suspected complications (e.g., obstruction, abscess, or perforation. In contrast, MRE avoids ionizing radiation, provides superior soft-tissue contrast, and can incorporate multiparametric readouts such as diffusion-weighted imaging and contrast-enhanced sequences, rendering it well suited for longitudinal surveillance, especially in younger patients. Clinically, both techniques are used for initial phenotyping and delineation of disease extent, monitoring inflammatory activity and complications, characterizing strictures (including inflammatory versus fibrotic components), assessing treatment response, and supporting perioperative planning and postoperative recurrence surveillance. Despite these strengths, conventional interpretation remains largely feature-based and semi-quantitative and is influenced by reader expertise, acquisition protocols, and contrast timing—limiting cross-center reproducibility. Moreover, semi-quantitative scoring does not adequately capture spatial heterogeneity, particularly in strictures where inflammation and fibrosis often coexist, resulting in ambiguous thresholds and inter-reader disagreement. Manual assessment is also ill-equipped to integrate high-dimensional information across sequences, phases, and combined bowel-wall–mesenteric phenotypes, constraining its utility for predicting complex outcomes such as treatment failure, relapse, and surgical endpoints (76). Collectively, these limitations highlight the need for more reproducible and verifiable quantitative frameworks, providing a clear clinical rationale for developing AI-enabled radiomics biomarkers in CD.

## 4 AI-radiomics: a methodological framework from imaging phenotypes to imaging biomarkers

Radiomics converts medical images—such as CT, MRI, PET, and ultrasound—into mineable high-dimensional data by extracting large numbers of quantitative features that are often imperceptible to the human eye (e.g., shape, intensity, texture, and higher-order descriptors) (13). By linking these features to clinically relevant endpoints, radiomics has the potential to shift clinical decision-making from experience-based judgment toward data-driven inference (77).Machine learning (ML) is a major branch of artificial intelligence that learns predictive patterns from data to perform classification, regression, or risk stratification (78). Deep learning (DL) is a subset of ML that uses deep neural networks—most commonly convolutional neural networks (CNNs)—to learn hierarchical representations directly from raw images, thereby reducing reliance on hand-crafted feature engineering (79). In radiomics research, commonly used ML algorithms can be broadly grouped into: (i) linear/regularized models (e.g., logistic regression, LASSO/Elastic Net), which are relatively simple and interpretable and can mitigate overfitting in high-dimensional, small-sample settings through regularization; (ii) kernel-based methods (e.g., support vector machines[SVM]), which often maintain strong discriminative performance with limited sample sizes and can model non-linear decision boundaries via kernel functions; (iii) tree-based and ensemble approaches (e.g., decision trees, random forests, and gradient-boosted decision trees/GBDT), which capture non-linear relationships and feature interactions, are less sensitive to feature scaling, and typically deliver strong predictive performance; and (iv) neural network methods, among which DL models (e.g., CNNs) are particularly well suited for tasks such as segmentation, detection, and end-to-end modeling by learning feature representations directly from images, although they generally require larger datasets and are more susceptible to domain shift, underscoring the need for robust external validation (80, 81).

In CD, a typical radiomics proceeds as follows: lesions are segmented on CTE or MRE to define regions of interest (ROI) or volumes of interest (VOI), using manual, semi-automated, or DL-based automated approaches; quantitative features are then extracted using software packages (e.g., PyRadiomics) or learned via DL. After feature selection or dimensionality reduction (e.g., LASSO), models are trained and evaluated on independent training and test sets, with performance commonly summarized using the area under the receiver operating characteristic curve (AUC). Where appropriate, decision curve analysis (DCA) is additionally used to estimate potential clinical net benefit across a range of decision thresholds (82).

### 4.1 Data acquisition and standardization: imaging parameters determine feature comparability

Radiomics assumes that histopathologic differences can be captured and quantified on imaging in a stable and reproducible manner. In practice, however, variability in image acquisition and reconstruction often alters intensity distributions and texture statistics to a degree that can exceed disease-related signals, resulting in radiomic features that are not directly comparable across centers—even when derived from the same lesion (83, 84). Key sources of variability include scanning and reconstruction parameters (e.g., slice thickness, convolution kernel/reconstruction algorithm, noise-reduction strategies, magnetic field strength, sequence settings, and b-values), as well as contrast-enhancement factors (e.g., dose, injection rate, and arterial/venous/delayed timing) (85). Accordingly, study protocols should be harmonized as much as possible at the design stage, and manuscripts should transparently report the critical acquisition and reconstruction parameters. During preprocessing, standard steps such as resampling, gray-level discretization, and intensity normalization can further reduce non-biological variability (86). In multi-center settings, feature-level batch-effect correction methods (e.g., ComBat) may also be considered to mitigate systematic center-specific shifts (87). Notably, while ComBat and related approaches have been shown to improve inter-site consistency and model generalizability in multi-center MRI radiomics, the magnitude of benefit varies by task and feature class (87). Therefore, harmonization should not be viewed as a substitute for external validation; a more rigorous approach is to implement both in parallel as complementary components of a clinically translatable pipeline.

### 4.2 ROI/VOI segmentation: a major hidden noise source

Because radiomics involves high-dimensional feature extraction, even subtle differences in ROI/VOI boundaries can lead to substantial variation in shape descriptors, texture metrics, and intensity statistics (88). Segmentation therefore represents a major bottleneck for reproducibility. Manual segmentation offers flexibility but is highly operator-dependent. Semi-automated and fully automated methods can improve efficiency and reduce subjective variability; however, they are susceptible to domain shift under real-world conditions—such as differences in scanners and protocols, contrast timing, or bowel peristalsis—which may introduce systematic errors (79, 89). Importantly, prior studies indicate that radiomic feature classes differ markedly in their sensitivity to segmentation perturbations, with direct implications for model stability and predictive performance (90). Accordingly, beyond simply reporting the segmentation approach, radiomics studies should quantify and disclose feature robustness to segmentation variability—for example, using intraclass correlation coefficients (ICC) or concordance correlation coefficients (CCC), as well as repeat-segmentation sensitivity analyses—and, when feasible, incorporate dual-reader annotation or consensus labeling as a reference standard (90). For automated segmentation, it is also advisable to report not only geometric overlap metrics (e.g., Dice) but, critically, (i) feature-level agreement relative to manual delineation and (ii) consistency of downstream task performance, since these measures more directly reflect the clinical impact of segmentation uncertainty (91).

### 4.3 Feature extraction and selection: prioritize reproducibility before predictivity

Two recurrent challenges in radiomics studies arise from (i) inconsistencies in feature definitions and computational details and (ii) statistical instability stemming from the “high-dimensional, low-sample-size” setting, where the number of extracted features far exceeds the number of subjects (92). To improve cross-study reproducibility, feature computation should adhere as closely as possible to the Image Biomarker Standardization Initiative (IBSI) guidelines, and authors should transparently report image preprocessing procedures, discretization parameters, and the software package and version used. This level of reporting helps minimize non-comparability caused by “same name–different definition” or “different name–same definition” issues across studies (93). For feature selection, robustness and redundancy control should be prioritized. A recommended approach is to perform stability filtering before model building—e.g., retaining only features that remain consistent under test–retest conditions or segmentation perturbations—followed by correlation-based filtering and dimensionality reduction/selection (such as LASSO, mRMR, or PCA) to limit collinearity and overfitting (94). Critically, feature selection and hyperparameter tuning must be conducted strictly within the cross-validation loop. Performing feature selection on the full dataset before data splitting inevitably introduces information leakage and can substantially inflate estimated model performance (95).

### 4.4 Modeling and validation: discrimination is not the endpoint; generalization and calibration are

Many radiomics models demonstrate strong performance during internal validation yet exhibit substantial degradation in external cohorts, a pattern that largely reflects overfitting and distribution shift between datasets (96). To mitigate overfitting, studies should adopt a rigorous training/validation/test split, or preferably use nested cross-validation: the inner loop is used for feature selection and hyperparameter tuning, whereas the outer loop provides an unbiased estimate of generalization performance (97). When reporting results, it is essential to clearly distinguish performance in the development set from that in an independent test set. Model evaluation should also extend beyond discrimination to include calibration and clinical utility. Discrimination can be summarized using metrics such as AUC or PR-AUC, calibration can be assessed with calibration curves and the Brier score, and clinical usefulness can be quantified using DCA to estimate net benefit across clinically relevant thresholds (98). This broader evaluation framework helps avoid models that are statistically impressive but difficult to translate into actionable clinical decisions.

### 4.5 Interpretability and clinical usability: from ‘works’ to ‘useful’

Clinical translation of imaging AI models depends not only on performance metrics, but also on whether clinicians can understand the output and use it smoothly in real workflows. From an interpretability standpoint, conventional machine-learning models can often be explained by reporting feature importance or using methods such as SHAP, which show how specific variables push risk estimates up or down (99). In contrast, deep learning models are commonly interpreted with visualization tools such as Grad-CAM, which highlight image regions that contribute most to a prediction (100). However, visually convincing heatmaps should not be mistaken for mechanistic insight. A more rigorous strategy is to compare explanation outputs with established imaging signs and known pathobiology and verify that the same explanation patterns remain consistent in external cohorts (101). In practice, interpretability is often most useful for detecting bias, understanding failure cases, and supporting clinician–patient communication rather than providing a definitive biological mechanism. With respect to usability, models should produce outputs that are directly actionable—for example, clear probability thresholds or clinically meaningful low-/intermediate-/high-risk categories. They should also state practical requirements up front: which CTE/MRE sequences and phases are needed, whether segmentation is required, expected runtime and computing needs, and common failure modes (e.g., motion, poor distension, protocol mismatch) (102). Finally, the model’s role in the clinical pathway should be clearly defined—for instance, whether it is intended to complement imaging scores and biomarkers such as fecal calprotectin and CRP to guide treatment escalation, follow-up interval adjustment, or timing of surgical evaluation. Ultimately, radiomics-based imaging biomarkers will have true translational value only if multi-center external validation—and ideally prospective studies—demonstrate that using the model improves decision-making and leads to measurable benefits in patient outcomes.

## 5 AI-radiomics enables quantifiable imaging biomarkers for precision management of CD

For an at-a-glance overview, **Table 1** summarizes representative AI-radiomics studies across major clinical applications in CD (not intended to be exhaustive) (16, 103–111).

**Table 1:** Representative CTE/MRE AI-radiomics models and performance across major clinical applications in Crohn’s disease.

Application	Objective	Dataset	Extraction of radiomic features	Key features	Best model	Performance (AUC)	Comparison	Reference
Diagnosis	CD vs. Non-CD	135patients (70 CD, 65 Non-CD)	3D Slicer + PyRadiomics + LASSO	MRE radiomic features + 8 Clinical Features (age, gender, weight, height, BMI Z-score, CRP, ESR, and fecal calprotectin)	Clinical Radiomics Model	0.98 (Integrated model) 0.95 (Radiomic model) 0.85 (Clinical model)	Superior to radiologists (AUC 0.837–0.881)	(103)
Differential Diagnosis	CD vs. ITB	330 patients(CD or ITB) SMOTE dataset(n=285) Validation dataset1(n=224) Validation dataset2(n=106)	ResNet50 + LASSO	CTE arterial/venous phase features	Arterial-Venous combined DL radiomics model	0.885 (SMOTE dataset) 0.877 (Validation dataset1) 0.800 (Validation dataset2)	Superior to the handcrafted radiomics model in same phase images	(104)
		160 patients(93 CD,67 ITB) Training cohort (n=114) Test cohort (n=49)	ITK-SNAP + PHIgo Workstation + GBDT	9 radiomic features(CTE) + 2 clinical factors(T-SPOT, small-intestine segmental lesions)	Clinical Radiomics Model	0.96 (Training cohort) 0.93 (Test cohort)	Superior to Clinical Model(AUC 0.91,0.90) or Radiomic model(AUC 0.90,0.86)	(105)
	CD vs. PIL	Center1 120 patients(51 CD, 69 PIL) Training set (n=84) Internal validation set (n=36) Center2 54 patients (30 CD and 24 PIL) External validation set (n=54)	ITK-SNAP + PyRadiomics + LASSO	13 radiomic features(CTE) + 4 clinical factors(frequency of defecation,intestinal wall thickness, comb sign and enhanced pattern)	Conbined model	1.00 (External validation set)	Superior to Radiomic model (AUC 0.98)	(106)
Activity Assessment	Active vs. Remission	107 CD Training set (n=84). Validation set (n=23)	nnU-Net + PyRadiomics + LASSO	12 radiomic features(CTE)+ IBDQ + 2lab data(ESR, fecal calprotectin)	Conbined model	0.969 (Training set) 0.877 (Validation set)	Superior to Clinical Model , Radiomic model, IBDQ	(107)
Fibrosis Assessment	Non-Mild vs. Moderate-Severe	167CD (3 centers) Training cohort (n=87) Validation cohort (n=80)	MITK + An internal software +LASSO	4 radiomic features(CTE)	Radiomic model	0.888(center1,Training set 1) 0.816(center1,Validation set 1) 0.724(center2,Validation set 2) 0.750(center3,Validation set 3)	Superior to radiologists(AUC 0.508, 0.567)	(108)
		235CD (3 centers ) Training cohort(n=114) Total test cohort(n=121)	ResNet + t-SNE	Deep learning features	Deep learning model(DLM)	0.828(Training cohort ) 0.811(Total test cohort) 0.808(Test cohort 1) 0.839(Test cohort 2)	1.Comparable to RM, faster processing 2. Superior to radiologists(AUC 0.579, 0.646)	(109)
Treatment Response	Mucosal Healing	246CD (3 centers) Training cohort (n=141) Testing cohort (n=61) Validation cohort(n=44)	3D ResNet101 + LASSO	Radiomics features + Deep Larning features	DLR model	0.948 (Training cohort ) 0.889 (Testing cohort ) 0.938 (Validation cohort )	Superior to RM(AUC 0.817,0.797,0.822)and DL(0.915,0.871,0.878)	(110)
Surgical Risk	1-Year Surgery Risk	201CD Training cohort(n=135) Testing cohort (n=69)	Pyradiomics + Boruta + XGBoost	Creeping fat radiomic features (CTE)	Intestinal-creeping fat combined radiomic model	0.916 (Training cohort) 0.822 (Testing cohort)	Superior to the single intestinal or creeping fat radiomic models	(111)
	Surgery Requirement	73CD(21 high risk,52 low risk)	3D Slicer	8 radiomic features(MRE) + sMARIA + clinical variables	Combined model	0.83	Superior to radiomics alone (AUC 0.74)	(16)

CD, Crohn's Disease; Non-CD, Non-Crohn's Disease controls; ITB, Intestinal Tuberculosis; PIL, Primary Intestinal Lymphoma; MRE, Magnetic Resonance Enterography; CTE, Computed Tomography Enterography; AUC, Area Under the Receiver Operating Characteristic Curve; LASSO, Least Absolute Shrinkage and Selection Operator; GBDT, Gradient Boosting Decision Trees; ResNet, Residual Neural Network; nnU-Net, No New-Net; t-SNE, t-distributed Stochastic Neighbor Embedding; XGBoost, eXtreme Gradient Boosting; SMOTE, Synthetic Minority Over-sampling Technique; DL, Deep Learning; DLR, Deep Learning Radiomics; RM, Radiomics Model; IBDQ, Inflammatory Bowel Disease Questionnaire; CRP, C-Reactive Protein; ESR, Erythrocyte Sedimentation Rate; sMARIA, simplified Magnetic Resonance Index of Activity; BMI, Body Mass Index.

### 5.1 Radiomics can improve consistency in diagnosis, phenotyping, and differential diagnosis

Establishing a definitive diagnosis is the first and most pressing clinical need in CD. Yet, at initial presentation, clinicians often face several challenges: small-bowel involvement may be difficult to assess comprehensively, manifestations can be atypical, and differentiation from ulcerative colitis (UC), intestinal tuberculosis (ITB), primary intestinal lymphoma (PIL) and other mimics is frequently problematic. Traditional image interpretation remains highly experience-dependent, with limited inter-reader and inter-center consistency. Emerging evidence suggests that CTE/MRE-based radiomics and deep learning models can provide strong discriminatory performance for noninvasive diagnosis and differential diagnosis of CD. For example, in the setting of suspected ileal CD, one study trained a linear support vector machine (SVM) using radiomic features extracted from non-contrast T2-weighted MRE of the ileum and adjacent mesenteric fat, achieving diagnostic performance superior to that of experienced radiologists; model performance improved further when combined with clinical variables (112). A recurring pattern across high-performing diagnostic models is the deliberate inclusion of extra-mural regions—particularly mesenteric/perienteric compartments—within the ROI/VOI (113). This “multiregional” strategy is also common in CD differential diagnosis. In the differential diagnosis model of CD and ITB by Tong Gong et al., radiomic features were extracted from multiple targets (the bowel-wall lesion, the largest lymph node, and the pericecal/perilesional region) and integrated with clinical and endoscopic information, while maintaining a high AUC in the validation cohort (114). Collectively, these findings support the premise that regions beyond the bowel wall (e.g., lymph nodes and perienteric inflammatory zones) contribute clinically meaningful information for differential diagnosis.

These mural imaging manifestations—bowel-wall thickening, hyperenhancement, ulceration, and luminal narrowing—ultimately arise from a common set of histopathologic processes, including vasodilation with increased vascular permeability, interstitial edema, inflammatory cell infiltration, and, in chronic stages, fibrotic remodeling (115). Because these mechanisms can occur across CD, ITB, PIL, and even severe infectious enteritis, models that focus solely on mural features are prone to being trained as “inflammation intensity classifiers,” rather than truly discriminative diagnostic tools (35). In contrast, extra-mural compartments—such as mesenteric/perienteric fat, mesenteric vasculature, regional lymph nodes, and the perilesional inflammatory zone—encode whether inflammation has breached the mucosa–submucosa boundary to generate a transmural and extra-mural response. They also reflect whether immune-cell recruitment is preferentially routed through vascular versus lymphatic pathways and whether adjacent tissues have undergone sustained immunometabolic reprogramming, particularly within mesenteric adipose tissue. These dimensions are often more informative for capturing disease-specific biology and thereby improving differential diagnosis. For example, UC is largely confined to superficial mucosal inflammation and typically lacks the prominent extra-mural adipose remodeling, comb sign, and penetrating phenotypes (sinus tracts/fistulas) that characterize the “transmural–mesenteric reaction” continuum in CD (116). In ITB, necrotic lymphadenopathy is more characteristic, whereas PIL more often shows tumor-driven extra-mural infiltration with bulky, conglomerate nodal disease and mass effect, rather than the inflammatory vascular–adipose response pattern typical of CD (117, 118). Accordingly, when developing radiomics models for the initial diagnosis and differential diagnosis of Crohn’s disease, extra-mural information should be treated as a key input and jointly integrated with mural features to enable more discriminative, clinically meaningful inference.

### 5.2 Radiomics can quantify transmural inflammatory burden and stratify disease activity

As CD management shifts from symptom control toward treat-to-target(T2T) strategies, reliance on symptoms or a single inflammatory marker is often insufficient to accurately capture transmural inflammatory burden and overall disease activity (119). This transition underscores the need for reproducible, quantitative imaging tools to support risk stratification and longitudinal assessment. In a study published in Radiology, investigators quantified inflammatory activity in CD using normalized mucosal iodine density derived from dual-energy CTE and directly benchmarked this metric against histopathologic grading of active inflammation. The results showed that iodine density reliably discriminated bowel segments with versus without active inflammation, whereas clinical activity indices did not differ significantly across histopathology-defined groups (120). These findings underscore the greater objectivity and interpretability of quantitative imaging readouts for stratifying inflammatory activity.

To improve the precision of radiomics for assessing inflammatory activity in CD, three methodological considerations deserve emphasis. First, ROI/VOI definition should be aligned with the transmural inflammatory burden rather than confined to a single mural contour (114). Layered wall sampling, perienteric or mesenteric rings, and multiregional targets—such as mesenteric fat and other reactive compartments—can better capture the spatial organization of inflammation (121). Second, sequence selection should be guided by an understanding of the underlying inflammatory biology, prioritizing imaging readouts that reflect the major histopathologic consequences of active disease (121). During active transmural inflammation, IL-23/Th17– and NF-κB–driven immune-cell infiltration and inflammatory cascades increase tissue cellularity, making DWI/ADC a preferred readout of cellular burden (122). In parallel, these pathways promote endothelial activation and mediator release, increasing microvascular perfusion and permeability and exacerbating interstitial leakage; accordingly, contrast-enhanced metrics can serve as readouts of perfusion and permeability, including dynamic contrast-enhanced parameters and, when available, dual-energy iodine maps (123). In addition, inflammatory leakage and tissue injury increase free water content and mural edema, which are more sensitively captured on T2-weighted imaging. Third, comparability of enhancement phase and acquisition/reconstruction protocols should be enforced across cohorts to minimize phase-related bias and parameter drift that undermine feature stability and multicenter generalizability (124).

### 5.3 Radiomics supports component stratification and risk grading of mixed inflammatory–fibrotic strictures

A central challenge in managing CD-related intestinal strictures is that inflammation and fibrosis frequently coexist, yet the therapeutic implications are fundamentally different: inflammation-dominant strictures may respond to intensified anti-inflammatory therapy, whereas fibrosis-dominant strictures more often require endoscopic dilation or surgery (125, 126). Although conventional imaging signs—such as wall thickness, T2-weighted edema, and early versus delayed enhancement patterns—provide useful clues, they rarely enable a quantitative estimate of the “fibrotic fraction,” leaving a substantial clinical gray zone (127). Consistent with this limitation, expert consensus statements emphasize that reliable assessment of fibrotic burden remains a major unmet need in routine practice (9). Emerging evidence supports the feasibility of AI-radiomics for compositional stratification of strictures. For instance, Chirra and colleagues extracted radiomic features from MRE of terminal ileal strictures and demonstrated the ability to identify severe histologic fibrosis and severe histologic inflammation; performance improved further when radiomics was combined with radiologist-based visual scoring, suggesting complementary value for stricture characterization (14).

However, for these approaches to truly inform clinical decision-making, interpretability and cross-center generalizability require that model features be anchored to testable immuno–fibrotic biology. Specifically, fibrotic programs—exemplified by TGF-β–driven remodeling and heightened macrophage–fibroblast crosstalk—are expected to map onto a composite imaging signature characterized by reduced tissue water content, delayed contrast retention (reflecting altered interstitial space), and an increased structural damage burden. In contrast, transmural inflammation dominated by the IL-23/Th17–TNF axis more commonly aligns with edema, hyperperfusion, and diffusion restriction. On this basis, radiomic features can be organized into three biologically oriented modules: (i) texture features capturing tissue densification and relative homogeneity, (ii) enhancement kinetics features reflecting early perfusion and delayed retention, and (iii) morphologic features quantifying structural damage burden, such as stricture length and upstream dilation (128). The resulting models can output probabilities for fibrotic and inflammatory components, together with an uncertainty estimate, thereby explicitly flagging the clinical “gray zone” of mixed inflammatory–fibrotic strictures.

### 5.4 Radiomics can predict treatment response at baseline and early follow-up to support personalized therapy

Within a T2T framework, imaging is increasingly expected to address not only “how inflamed the bowel is today,” but more decision-relevant, forward-looking questions: after initiating infliximab (IFX), is a patient at risk for primary non-response or early treatment failure; will secondary loss of response (SLR) emerge during maintenance; and when should clinicians consider dose optimization, therapeutic drug monitoring (TDM), or switching to an alternative mechanism of action (129). Emerging evidence supports the feasibility of radiomics for these tasks. For example, a Radiology and Medical Imaging (Radiol Med) 2024 model integrated CTE-derived radiomic signatures with body-composition metrics to predict the risk of IFX treatment failure (130). Similarly, a 2024 European Radiology/Insights into Imaging study proposed a combined model incorporating VAT radiomics and bowel-wall imaging features to improve prediction of IFX response, supporting the concept that “lesion phenotype and body-composition phenotype” provides a more informative input space than bowel-wall features alone (131).

Even more actionable for longitudinal management is a dynamic strategy based on delta-radiomics (Δ-radiomics) (132). In most protocols, the “early” window aligns with post-induction reassessment—approximately 12–14 weeks—and Δ-radiomics quantifies the change in imaging-derived features between baseline (pre-treatment) and the end of induction (week 12–14) to capture treatment trajectories. In a 2025 longitudinal CT delta-radiomics study, temporal changes in VAT-derived features identified patients at high risk for subsequent SLR even among those who had achieved clinical remission, underscoring that “symptom improvement” does not necessarily translate into “low maintenance-phase risk” (132). Accordingly, an adverse Δ signal at 12–14 weeks may serve as an early trigger for proactive intervention, including intensified monitoring, earlier TDM, dose escalation, or timely mechanism switching. To ensure that such models meaningfully support T2T pathways, reporting should extend beyond discrimination (AUC) to include calibration and DCA, alongside external validation and robustness assessments across protocol heterogeneity, thereby moving imaging from static status assessment toward a tool for early strategy refinement and dynamic disease management.

### 5.5 Radiomics integrates bowel wall–mesentery signals to assess complications and surgical risk

Hospitalization and surgery in CD are most often driven by penetrating complications (abscesses, sinus tracts, fistulas) and recurrent stricturing, yet routine image interpretation often remains binary—whether a complication is present—offering limited guidance on who will progress, when to intervene, and whether recurrence is likely after surgery (133). These outcomes typically arise along a continuum of transmural inflammation extending across the bowel wall–perienteric fat–mesentery axis, such that actionable risk information is frequently encoded in extramural features (e.g., abscess location/size, the course and caliber of sinus tracts/fistulas, and the surrounding inflammatory “fat field”) (134). This is exemplified by intra-abdominal abscesses, where contrast-enhanced CT features including non-perienteric location, maximal diameter, and sinus-tract width correlate with subsequent need for drainage or surgery and can be combined into a high-performing score (AUC 0.89) (135). These observations suggest that ROIs should extend beyond the bowel wall to include the abscess wall, sinus/fistula trajectory, and perilesional inflammatory zone. Consistent with this “bowel wall–mesentery” paradigm, a CTE radiomics study that segmented both inflamed bowel and creeping fat showed incremental prognostic value for 1-year surgery risk, with the combined model outperforming either compartment alone (test AUC 0.882) (111). Similar approaches have been reported for longer horizons (e.g., 10-year surgical risk stratification; AUC 0.83) and for perioperative prediction, where incorporating radiomic signatures from both the intestinal lesion and peri-intestinal mesenteric fat improved early anastomotic recurrence risk assessment relative to clinical–radiologic factors alone (136, 137).

## 6 Integration with immune biomarkers and multi-omics: making ‘alignment’ operational

In CD, AI-radiomics is often more sensitive than conventional visual assessment to transmural inflammatory burden, mesenteric reactions, the morphologic burden of strictures, and enhancement kinetics. However, a key limitation of radiomics alone is that it does not directly reveal the underlying molecular drivers. By contrast, immune biomarkers and multi-omics profiling—spanning proteomics, transcriptomics, immune-cell states, metabolomics, and microbiome data derived from blood, stool, and tissue—can interrogate inflammatory pathways, cellular composition, and metabolic axes, yet often lack spatial localization and are constrained by sampling bias (e.g., endoscopic biopsies are local, blood reflects systemic signals, and stool is luminal) (138). Therefore, integrating AI-radiomics with immunologic and multi-omics data is, in essence, a strategy to connect spatial heterogeneity with molecular mechanisms: imaging defines reproducible spatial disease phenotypes, multi-omics provides mechanistic interpretation of the pathways and cellular ecosystems underlying these phenotypes, and molecular readouts, in turn, enhance the interpretability and clinical credibility of imaging biomarkers.

It is worth emphasizing that the value of ML in CD research extends beyond outcome prediction to include mechanism discovery and biomarker identification. At the omics level, a typical workflow first applies feature selection and dimensionality reduction to high-dimensional datasets—such as transcriptomics, proteomics, metabolomics, and the microbiome (e.g., LASSO, random forests, gradient boosting, and SVM-RFE)—and then integrates pathway enrichment, cross-omics association analyses, network modeling, or module-based approaches (e.g., correlation networks to identify pathway modules and hub molecules). This strategy yields interpretable outputs, including pathway activity scores, molecular subtypes, or panels of key hub molecules and/or microbe–metabolite combinations. Representative studies applying ML to multi-omics data for CD mechanism discovery and biomarker identification are summarized in **Table 2** (139–143).These ML-derived mechanistic signals, in turn, provide anchors for imaging: they can be used to test whether specific imaging phenotypes (e.g., transmural edema/hyperenhancement, diffusion restriction on DWI, mesenteric fat reaction, or reduced distensibility in strictures) map onto defined inflammatory axes (such as TNF or IL-23/Th17), immune-cell ecologies, or fibrotic remodeling programs; alternatively, mechanistic indices can be set as learning targets for imaging models so that imaging phenotypes become not only predictable but also interpretable and biologically traceable.

**Table 2:** Representative machine learning–based multi-omics studies for mechanism discovery and biomarker identification in Crohn’s disease.

Study	Data (sample+ omics)	ML methods	Key biomarkers	Mechanistic implication
Preto et al. (2025, *J Crohn’s Colitis*) (139)	SPARC IBD(hundreds of UC/CD patients); Genomics + Gut Biopsy Transcriptomics + Plasma Proteomics	XGBoost (UC vs CD classifier) + MOFA (patient stratification; CD/UC subgroups)	Task 1 (CD vs UC): IL12B, LY96, CCL20, FGF19/INSL5; IGKV6D-21, GALNT6, TBX3. Task 2 (UC severity): IL17A, TGFA, REG1B; DLD, EMILIN2. Task 3 (CD inflammatory subgroups): CXCL9, HLA-DRA, SERPINA3, NOS2; TSBP1-AS1, HLA-related transcripts.	Reveals CD-relevant molecular programs underlying diagnosis and subgrouping, mainly involving IL-12/IL-23–Th1/Th17 inflammatory signaling, innate immune activation, and immunometabolic/endocrine pathways.
Lei et al. (2025, Frontiers in Molecular Biosciences) (140)	Urine; targeted metabolomics (49 central-carbon metabolites); UC/CD/controls (n=95); UHPLC-MS/MS quantification.	DT(Decision Tree)、Ridge(Ridge regression classifier)、SVM、NN(Neural Network)、LightGBM、RF(Random Forest)	Task 1(CD vs control): xylose, L-fucose, citric acid Task 2(UC vs control): xylose, isocitric acid, fructose, L-fucose, GlcNAc, glycolic acid.	IBD subtype–specific perturbations in central carbon metabolism (TCA cycle + sugar/hexosamine pathways) underpin noninvasive urinary biomarker panels.
Chen et al. (2025, Scientific Reports) (141)	GEO GSE95095 intestinal tissue microarray (24 CD / 12 controls) + external validation GSE83448 (19 CD / 14 controls); ubiquitination-gene set intersection; RT-qPCR in LPS-Caco-2 (± TNBS mouse tissue).	LASSO (10-fold CV, glmnet) + Random Forest feature selection; combined with PPI hub selection (Cytoscape bottleneck/eccentricity) and ROC validation.	Ubiquitination-related genes: UBE2R2 and NEDD4L.	Links ubiquitination dysregulation to the CD immune microenvironment, with reduced M2 macrophage infiltration and opposite correlations of UBE2R2/NEDD4L with M2 infiltration; pathway analyses implicate autophagy (UBE2R2) and Wnt/calcium/lysosome signaling (NEDD4L).
Metwaly et al. (2020, *Nature Communications*) (142)	Longitudinal fecal 16S rRNA microbiome profiles in a CD–HSCT cohort, plus untargeted metabolomics (UPLC/TOF-MS) in humanized gnotobiotic mice colonized with donor microbiota, linked to clinical activity/outcomes.	Random Forest with 10-fold cross-validation for classifying disease activity/HSCT outcome; PLS-DA for metabolite discrimination/feature selection (mouse metabolomics).	Functional biomarker signatures: enrichment of sulfur transport/sulfur metabolism KEGG modules, plus a bacteria–metabolite interaction network highlighting sulfur-related features.	Supports a mechanistic link between CD activity and sulfur-metabolism–centered host–microbiome pathways, suggesting that functional (pathway-level) dysbiosis, rather than taxonomic composition alone, may drive inflammation and relate to disease outcome.
Kim et al. (2023/2024, Microorganisms) (143)	Fecal 16S rRNA microbiome; CD (n=671), UC (n=114), healthy controls (n=1462).	sPLS-DA (100× downsampling with age/sex/sample-size matching; train/test splits); two binary models (IBD vs HC; CD vs UC).	Task 1 (IBD vs HC): Lactobacillus/Levilactobacillus, Streptococcus, Actinomyces/Rothia, Parabacteroides/Alistipes, Prevotella (representative genera). Task 2 (CD vs UC): Sediminibacterium, Undibacterium/Lautropia, Ruminococcus_torques_group, Lachnospiraceae FCS020_group, Oscillospiraceae MKA214 (representative genera)	A compact fecal genus signature distinguishes IBD from health and separates CD from UC, supporting disease-specific dysbiosis patterns as noninvasive diagnostic biomarkers.

However, to date, high-quality CD studies that tightly pair CTE/MRE radiomics with multi-omics remain relatively scarce. This likely reflects several constraints: difficulties in achieving segment-level spatial alignment and time-window matching between imaging and biospecimens; the lack of unified and reliable reference standards for key phenotypes, particularly fibrosis; the high dimensionality of both imaging and omics features, which necessitates larger cohorts and rigorous cross-center harmonization; substantial domain shifts introduced by differences in scanners and protocols; and limited reproducibility due to ROI segmentation variability and feature robustness issues. Against this backdrop, a longitudinal multi-omics study published in Nature—focused on assessing disease activity in CD—provides a useful methodological template (144). In that multi-center cohort with approximately one year of follow-up, investigators collected biopsies at baseline colonoscopy, obtained blood samples at quarterly visits, and acquired stool samples every two weeks via home collection. They generated multi-omics data including metagenomics (MGX), metatranscriptomics (MTX), proteomics, metabolomics, and viromics. After unified quality control and standardized functional annotation, clinical activity indices (HBI/SCCAI) and fecal calprotectin were used as longitudinal “time anchors.” Multi-modal measurements were matched within an allowable window of ≤4 weeks, and cross-omics correlation and network modeling integrated microbial composition and activity, functional pathways, metabolites, and inflammatory readouts to identify pathway modules and potential molecular “hubs” associated with fluctuations in disease activity. Building on this framework, future radiomics–multi-omics integration could be implemented by incorporating CTE/MRE at key visits within the same follow-up schedule, performing segment-level ROI delineation of the bowel wall and mesenteric compartments, and extracting radiomic features that are time-matched to contemporaneous omics measurements. Two complementary integration strategies can then be pursued. A phenotype-driven approach starts from imaging: patients are first clustered according to radiology-derived spatial phenotypes, and multi-omics analyses are subsequently used to identify the corresponding immune and metabolic mechanisms. A mechanism-driven approach starts from biology: pathway activity scores are derived from omics data, and imaging models are then trained to predict these mechanistic readouts as “imaging surrogates.” Regardless of strategy, robust evaluation requires validation in independent cohorts and assessment of clinically meaningful endpoints, such as treatment response, relapse, and surgery-related outcomes.

## 7 Conclusion

This review is framed around the multi-network pathobiology of CD, encompassing genetic susceptibility, epithelial barrier dysfunction, microbial dysbiosis, immune–inflammatory activation, fibrotic remodeling, and mesenteric inflammation accompanied by adipose remodeling. Building on this mechanistic foundation, we discuss how these processes give rise to quantifiable imaging phenotypes on CTE/MRE and highlight the potential of AI-radiomics to translate such phenotypes into imaging biomarkers. These biomarkers may improve the reproducibility of diagnosis and differential diagnosis, enable more granular quantification of disease activity and transmural burden, and support earlier risk identification and more individualized management strategies for fibrostenotic stratification, treatment response prediction, and complication/surgical risk assessment. Importantly, imaging-derived phenotypes should be interpreted within a broader biological context: integrating radiomics with circulating and fecal biomarkers as well as multi-omics layers (e.g., transcriptomics, proteomics, immunophenotyping, metabolomics, and microbiome profiling) may yield a more mechanistically grounded, patient-specific disease signature by linking imaging patterns to their underlying immunopathological drivers. Overall, the value of AI-radiomics lies not in replacing conventional image interpretation, but in converting “visible yet hard-to-quantify” heterogeneity into verifiable, longitudinally trackable metrics that can be aligned with treat-to-target care and, ultimately, multimodal precision medicine.
